# MicroRNA Profiling of B Cell Subsets from Systemic Lupus Erythematosus Patients Reveals Promising Novel Biomarkers

**DOI:** 10.3390/ijms160816953

**Published:** 2015-07-27

**Authors:** Isabelle Duroux-Richard, Jimena Cuenca, Clara Ponsolles, Alejandro Badilla Piñeiro, Fernando Gonzalez, Christine Roubert, Roser Areny, Rosa Chea, Jacqueline Pefaur, Yves-Marie Pers, Fernando E. Figueroa, Christian Jorgensen, Maroun Khoury, Florence Apparailly

**Affiliations:** 1 INSERM, U1183, Institute of Regenerative Medicine and Biotherapy, University Hospital Saint Eloi, Montpellier 34295, France; E-Mails: clara.ponsolles@inserm.fr (C.P.); ym-pers@chu-montpellier.fr (Y.-M.P.); christian.jorgensen@inserm.fr (C.J.); florence.apparailly@inserm.fr (F.A.); 2 University of Montpellier, Montpellier 34090, France; 3 Laboratory of Nano-Regenerative Medicine, Faculty of Medicine, Universidad de Los Andes, Santiago 7620001, Chile; E-Mails: jcuenca@c4c.cl (J.C.); ABADILLA@uandes.cl (A.B.P.); fgonzalf@uc.cl (F.G.); ffigueroa@uandes.cl (F.E.F.); MKhoury@uandes.cl (M.K.); 4 Cells for Cells, Santiago 7620001, Chile; 5 Exploratory Unit, Sanofi R & D, Montpellier 34184, France; E-Mail: Christine.roubert@sanofi.com; 6 Hospital Félix Bulnes, Santiago 7510021, Chile; E-Mail: roser.areny@gmail.com; 7 Hospital Barros Luco, Santiago 8900085, Chile; E-Mails: rcheavi@gmail.com (R.C.); jacquelinepefaur@gmail.com (J.P.); 8 Clinical Department for Osteoarticular Diseases, University Hospital Lapeyronie, Montpellier 34295, France

**Keywords:** lupus, lupus nephritis, naive B cells, memory B cells, microRNAs

## Abstract

MicroRNAs control the differentiation and function of B cells, which are considered key elements in the pathogenesis of systemic lupus erythematosus (SLE). However, a common micro(mi)RNA signature has not emerged since published data includes patients of variable ethnic background, type of disease, and organ involvement, as well as heterogeneous cell populations. Here, we aimed at identifying a miRNA signature of purified B cells from renal and non-renal severe SLE patients of Latin American background, a population known to express severe disease. Genome-wide miRNA expression analyses were performed on naive and memory B cells and revealed two categories of miRNA signatures. The first signature represents B cell subset-specific miRNAs deregulated in SLE: 11 and six miRNAs discriminating naive and memory B cells of SLE patients from healthy controls (HC), respectively. Whether the miRNA was up or down-regulated in memory B cells as compared with naive B cells in HC, this difference was abolished in SLE patients, and *vice versa*. The second signature identifies six miRNAs associated with specific pathologic features affecting renal outcome, providing a further understanding for SLE pathogenesis. Overall, the present work provided promising biomarkers in molecular diagnostics for disease severity as well as potential new targets for therapeutic intervention in SLE.

## 1. Introduction

Systemic Lupus Erythematosus (SLE) is a chronic multisystem immune-mediated disease characterized by the production of antinuclear antibodies and periods of clinical remission and flares. Prognosis has improved in the western world, but higher incidence and poor survival are reported in certain ethnic groups. This is particularly the case with lupus nephritis (LN), a condition that has increased in the past decade, with higher mortality in Latin Americans, displaying poor renal outcome, and no change in mortality associated with current treatments. Despite decades of basic and clinical research, the mechanisms responsible for disease flares, tissue specificity, and patterns of organ involvement are still unclear.

Recent reports have suggested that epigenetic factors play a role in the onset and progression of SLE. These include DNA methylation, histone modifications, and micro(mi)RNA expression interfering with transcription and translation. MiRNAs are small non-coding RNA molecules known to regulate several processes including cell cycle and differentiation, apoptosis, and also immune responses. Several miRNAs related to B and T cell differentiation and survival, toll-like receptor (TLR) signaling, cytokine production, as well as innate and adaptive immune processes are known to participate in the pathogenesis of SLE and miRNA profiling studies have identified SLE-specific signatures associated with the disease [1,2]. A link between SLE and miRNA expression was first reported in 2007 by Dai *et al.* who identified a number of deregulated miRNAs in peripheral blood mononuclear cells (PBMCs) from SLE patients compared to healthy controls [3]. In the last years, a great deal of effort has been directed to identify specific patterns of miRNA expression related to SLE [4,5,6]. While all recent studies confirm the aberrant miRNA levels in SLE, a common miRNA signature has not yet emerged, mostly because cohorts of patients used for arrays exhibit variable patterns [7]. These dissimilarities highlight the importance of variability in ethnic background, severity and type of disease, as well as the type of biological samples analyzed and the limitation of performing gene expression studies in unfractionated, heterogeneous cell populations. In addition, while miRNA-mediated deregulation in SLE has been studied mostly in whole blood or isolated T cells, there are fewer studies that systematically report miRNA changes in lupus B cells.

Among the many immune cell types that have been involved in SLE, B-lymphocytes clearly play a central role in disease pathogenesis and progression. SLE is indeed characterized by abnormal B cell activation and differentiation to memory or plasma effector cells, associated with polyclonal B-cell hyper reactivity and formation of autoantibodies that target a variety of self-antigens. These autoantibodies are particularly fundamental in the pathogenesis of LN. Interestingly, miR-30a and miR-1246 control B cell hyperactivity through Lyn and EBF1 silencing, respectively, and their respective up- and down-regulation in B cells might contribute to SLE pathogenesis [8,9]. Among B cells, abnormal frequencies and functions of certain subsets, including disturbances of naive and memory B cells, have been reported in SLE patients [10]. Although distinct miRNA profiles have been reported in PBMC or purified CD19^+^ B cells of patients with SLE [5,6], none of the previous studies investigated miRNA expression in B cells, taking into account their functional heterogeneity.

The present work aimed at identifying a miRNA signature of purified B cell subsets from renal and non-renal severe SLE Latin American patients, a population known to express the severe complication of SLE. Using microarray technology, we identified a panel of 11 and six miRNAs that were differentially expressed between naive and memory B cells of SLE patients in comparison to healthy controls, respectively. One of these miRNAs (miR-29c) was associated with lupus nephritis and is reported here for the first time. In addition to representing potential new markers, these miRNAs may help to further understand the role of B cell subsets in SLE and to elucidate the pathological mechanisms of the disease.

## 2. Results

In an initial attempt to identify differentially expressed miRNAs in B cell subsets isolated from SLE patients of Latin American background, we performed microarray analyses comparing the expression levels of 782 miRNAs in Fluorescence-activated cell sorting (FACS)-sorted naive CD27^−^ and memory CD27^+^ B cells. Blood samples were collected prior to the bolus of corticosteroids and/or anti-inflammatory drugs from six SLE patients and four healthy controls (HC). The patients’ characteristics are presented in Table 1. All patients were relapsing and displayed active disease symptoms as assessed by British Isles *Lupus* Assessment Group (BILAG) and Systemic *Lupus* Erythematosus Disease Activity (SLEDAI) indexes. They were matched by gender, age, and ethnic background with HC.

**Table 1:** Clinical characteristics of SLE patients and healthy donors.

Characteristics	All Patients (*n* = 6)	SLE-LN (*n* = 4)	Controls
Age (years)	3611 ± 1275	3417 ± 1425	3000 ± 903
BILAG index	1456 ± 744	1683 ± 873	NA
SLEDAI index	1489 ± 597	1783 ± 479	NA
Medications	Pred 0 to 50 mg/day ± HCQ (200 mg/day)	Pred 0 to 20 mg/day ± HCQ (200 mg/day)	NA

*n*, patients number; SLE-LN, Systemic Lupus Erythematosus with lupus nephritis; Pred, prednisone; HCQ, Hydroxichloroquine; NA, not applicable.

**Figure 1:**
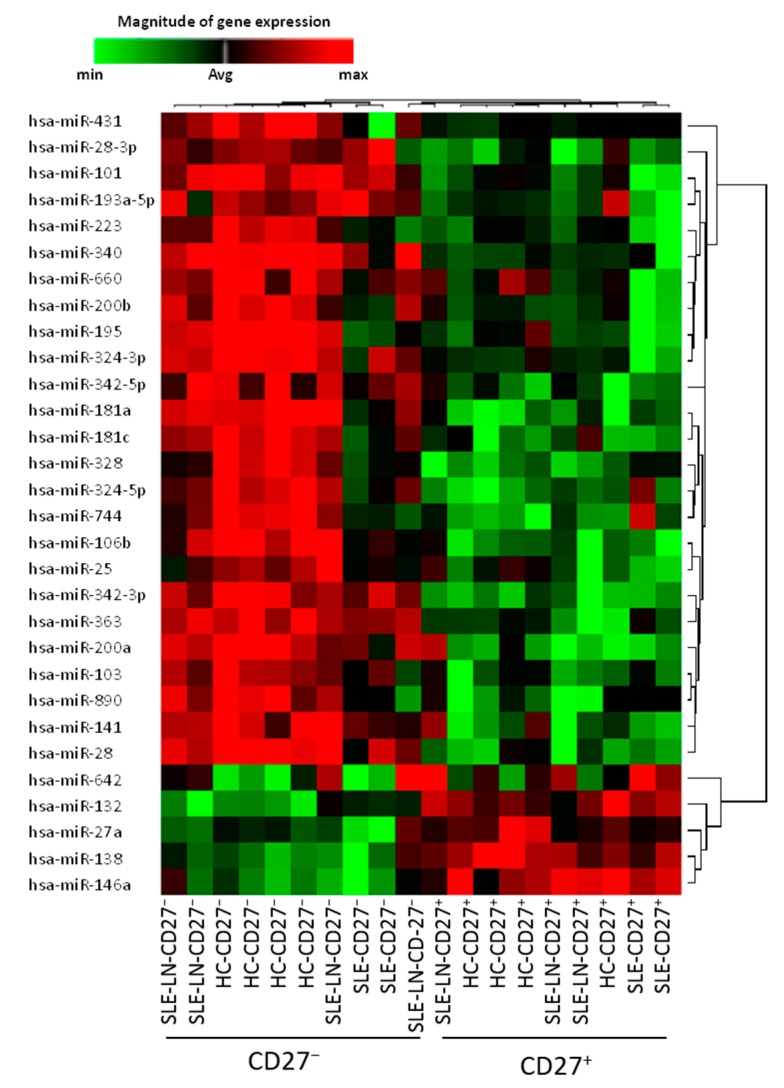
Hierarchical clustering of differentially expressed miRNAs in naive (CD27^−^) and memory (CD27^+^) B cells in healthy donors and SLE patients. The profiles of 30 microRNAs significantly differently expressed (*p* < 0.01) between CD27^+^ and CD27^−^ cell samples isolated from six SLE patients and four healthy donors were visualized using a supervised heatmap (average linkage and Pearson’s correlation). Relative miRNA expression was calculated using the comparative threshold cycle (CT) method. For normalization, the mean CT value of all miRNA targets was used. Dendrograms indicate the correlation between groups of samples and miRNAs. Samples are in columns and transcripts in rows. Each row represents a single miRNA and each column represents an individual sample. The heat map shows the corresponding relative miRNA expression levels rendered to green-red color scale (red being high expression level (max), green being low expression level (min) and black being absence of detection (Avg)).

A threshold of 1.8-fold differential expression and a *p*-value <0.01 were used to define significantly deregulated miRNAs. Based on the expression levels of the 782 assayed miRNAs in both B cell subsets, the unforced hierarchical clustering of the patients and HC showed that, during the active phase of the disease, lupus naive and memory B cells display similar transcriptional profiles as compared to their normal cell subset homologs (Figure 1). Independently of the clinical status of the individuals and/or disease severity, 30 miRNAs were found to be differentially expressed between both subsets, where four miRNAs were significantly increased in CD27^+^ memory B cells and 26 miRNAs were highly expressed in CD27^−^ naive B cells.

**Figure 2:**
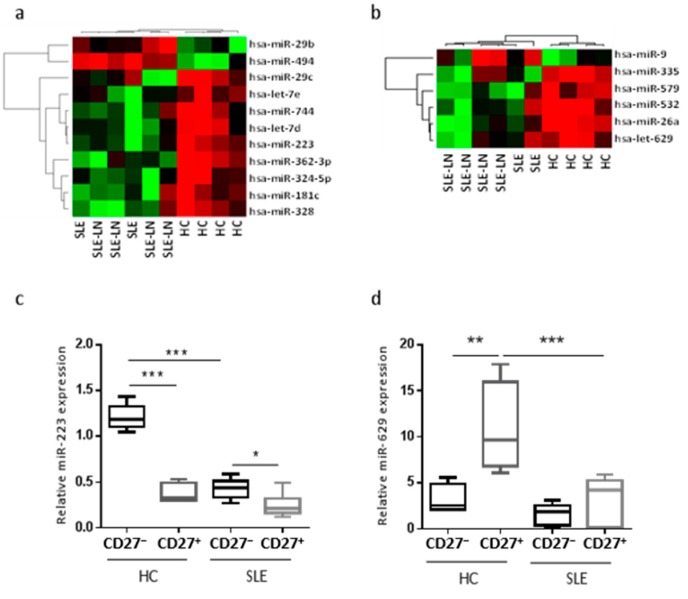
Unforced hierarchical clustering of each B cell subset discriminated SLE and LN patients from HC (healthy controls). The heat map shows relative levels of miRNA expression in a red (low relative expression) to green (high relative expression) scale. Dendrograms indicate the correlation between groups of samples and miRNAs. Samples are in columns and transcripts in rows. Each row represents a single miRNA and each column represents an individual sample. Heatmaps for naive (**a**) or memory (**b**) B cells are shown and are ordered by the disease (six SLE patients *versus* four HC), and show the expression for the 11 or six most deregulated miRNAs, respectively. Expression levels of miR-223 (**c**) or miR-629 (**d**) in naive and memory B cells. Results are expressed as mean ± SD of individual sample patients. * *p* < 0.05, ** *p* < 0.01, and *** *p* < 0.001, one-way ANOVA, followed by a Tukey’s multiple comparisons post-test.

However, the same unforced hierarchical clustering performed separately for each B cell subset discriminated SLE and LN patients from HC (Figure 2). The statistical analysis identified distinct gene expression profiles between SLE patients and HC, with subgroups of 11 (Figure 2a) and six (Figure 2b) miRNAs differentially expressed in naive and memory B cells, respectively. In the naive B cell subset, we observed two miRNAs significantly up-regulated (miR-29b and miR-494) and nine significantly down-regulated (miR-29c, -181c, -223, -324-5p, -328, -362-3p, -744, let-7d, and -7e) in SLE as compared with HC. As an example, the expression levels of miR-223 using real-time quantification by stem–loop Reverse transcription polymerase chain reaction (RT–PCR) were plotted (Figure 2c). The median expression of miR-223 was 2.8-fold higher in HC than in SLE and LN patients. In the memory B cell fraction from SLE patients, the expression levels of five miRNAs were decreased (miR-26a, -335, -532, -579, and -629) and only one miRNA was increased (miR-9), compared with HC samples. We found that miR-629 displayed the highest difference in memory B cells, being 4.2-fold more expressed in HC as compared with SLE patients. Expression levels of miR-629 using real-time quantification by stem–loop RT–PCR were plotted (Figure 2d). These results suggest that specific miRNAs differentially expressed in specific B cell subsets may be SLE disease-specific.

**Figure 3:**
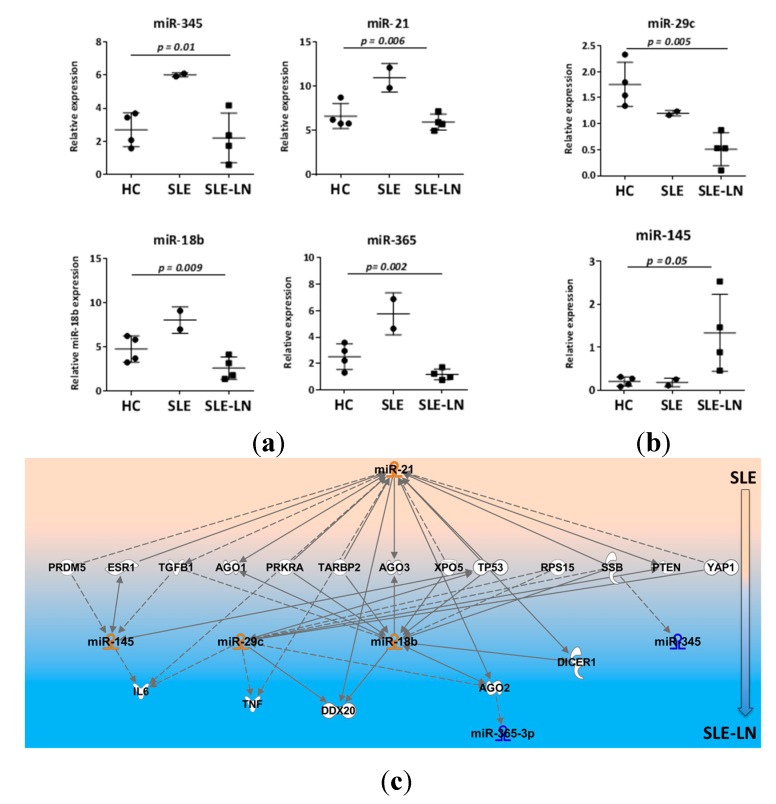
miRNAs discriminate SLE from SLE-LN patients independently on the B cell subset. Expression levels of miRNAs were detected in CD27^+^ and CD27^−^ B cells using real-time RT-PCR. For normalization, the mean CT value of all miRNAs analyzed and two housekeeping snoRNA genes (RNU48 and RNU44) were used. Each dot represents the mean value for CD27^−^ and CD27^+^ B cell subsets. (**a**) Expression of four miRNAs is similar for the SLE-LN and HC patients, but their expression is different between SLE and SLE-LN; (**b**) Expression of two miRNAs is different between SLE-LN and SLE or HC patients. Results are expressed as mean ± SD of individual samples of six SLE patients and four HC. *p*-values of the one-way ANOVA test are indicated on each graph; and (**c**) Hierarchical layout of the putative target genes predicted from miRNAs differentially expressed between SLE and SLE-LN was built using Ingenuity Pathway Analysis (IPA). Direct or indirect interactions are indicated by line or dotted line, respectively.

Among the 782 miRNAs measured using the high-throughput miRNA microarrays, we searched for miRNAs that significantly discriminate SLE from LN patients independently on the B cell subset. We observed that the expression levels of five miRNAs (miR-18b, -21, -29c, -345, and -365) seem to be decreased in LN patients as compared with SLE patients and one miRNA (miR-145) increased in renal (LN) forms of lupus as compared with non-renal SLE (Figure 3). Among the six miRNAs differentially expressed in LN patients, four miRNAs displayed an expression profile in LN patients that was different from SLE patients but similar to HC (Figure 3a), and two miRNAs displayed expression levels that were different from both HC and SLE patients (Figure 3b). These data suggest that a miRNA signature might be associated with disease type and may help to identify specific forms of lupus in Latin American.

Subsequently, we investigated the putative functional role of these six new LN-associated miRNAs. To this end, pathway enrichment analysis was performed using the web-based bioinformatics application IPA (Ingenuity Pathway Analysis) based on a list of *in silico* predicted target genes. A hierarchical layout was built with only miRNA/mRNA interactions displaying a high-predicted score and for which the correlation was experimentally observed in humans (Figure 3c). Such analysis showed that three out of the six miRNAs were known to be linked to SLE: miR-21 was described in several reports as deregulated in SLE [11] while miR-345 and -365-3p were reported abnormally expressed in kidney biopsies from patients with LN [12]. Our analysis also suggested that both miR-21 and miR-18b seem to function as molecular hubs able to directly or indirectly control numerous genes involved in B cell functions. Interestingly, reduced expression of miR-21 in SLE B cells was reported to contribute to B cell hyperactivity through PTEN (phosphatase and tensin homolog) silencing [11]. Pathway enrichment analysis indicated immune response, cell death and survival, as well as gene regulation and transcription among the top pathways potentially affected by the six miRNAs. Of note, miR-29c and miR-145, which are down- and up-regulated in B cells of SLE-LN patients and which displayed comparable expression levels in non-nephritis SLE and HC (Figure 3b), might be specific for the kidney-associated damages. Indeed, the inflammatory cytokines TNF (tumor necrosis factor), Interleukin-6, and BAFF (B-cell activating factor) are associated with B-cell kidney infiltration and have been shown to contribute to disease pathogenesis in patients with lupus nephritis [13,14,15]. They are also validated target genes for miR-29c [16,17] and therefore, reduced expression of miR-29c in B cells of nephritis SLE patients could possibly result in enhanced secretion of BAFF, TNF, and IL-6 in the kidneys of SLE-LN patients by locally infiltrating B cells. The increased expression of miR-145 in B cells of nephritis SLE patients could possibly contribute to reduced B cell proliferation and B cell lymphopenia, as observed in SLE-LN patients, since miR-145 is a negative regulator of B cell proliferation, invasion, and migration and is reduced in B cell malignancies [18].

**Figure 4:**
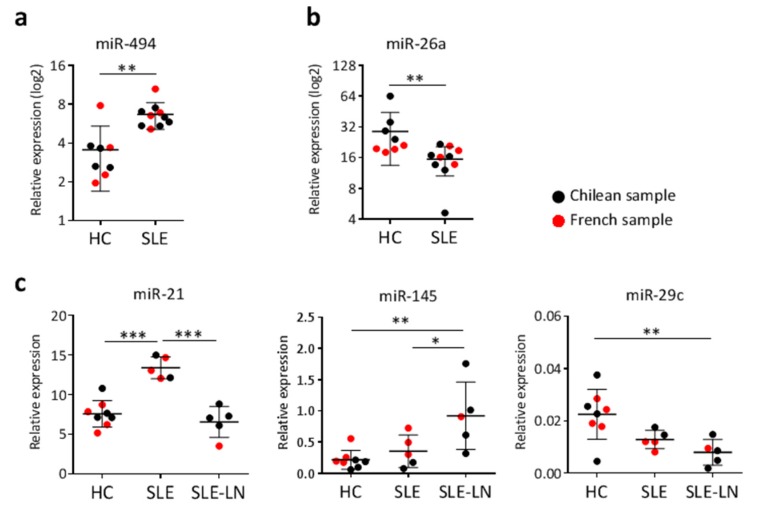
miRNA expression comparison between Chilean and French subjects. The expression level of five miRNAs was detected in CD27^+^ and CD27^−^ B cells using real-time RT-PCR. Each dot represents the mean value for CD27^−^ and CD27^+^ B cell subsets for each Chilean (black) and French (red) subject. (**a**) miR-494 expression in naive CD27^−^ B cells of SLE patients *versus* HC; (**b**) miR-26a expression in memory CD27^+^ B cells of SLE patients *versus* HC; (**c**) Expression levels of three different miRNAs that are representative of the three different expression profiles identified for discriminating SLE *versus* SLE-LN patients: miR-21, miR-145, and miR-29c. Results are expressed as mean ± SD of individual samples for five SLE (three French, two Chilean), five SLE-LN (one French, four Chilean), and eight HC (four French, four Chilean). * *p* < 0.05, ** *p* < 0.01, and *** *p* < 0.001 are plotted for one-way ANOVA followed by a Tukey’s multiple comparisons post-test, as large and small black lines, respectively.

Finally, to serve as a validation and extend our findings to other ethnicities, we selected a few miRNAs that are representative of each type of signature identified and quantified their expression levels in a small group of French SLE patients and healthy donors. In Figure 4, French samples are presented together with data from the Chilean groups for each corresponding miRNA investigated: miR-494 as miRNA discriminating naive CD27^−^ B cells of SLE patients *versus* HC (Figure 4a), miR26a as miRNA differentially expressed in memory CD27^+^ B cells of SLE patients *versus* HC (Figure 4b), and miR-21 and miR-145 as discriminating SLE from SLE-LN patients independently on B cell subsets, each one being representative of the three different profiles observed (Figure 4c). Overall, the expression levels of the five miRNAs tested in the French subjects were comparable to those found for the Chilean individuals. Although case numbers remain small, these findings suggest similarities between two different ethnic groups, at least for the five miRNAs analyzed so far.

## 3. Discussion

In the present study, we screened the expression profile of miRNAs in active SLE-affected patients, with and without nephritis, of an ethnically unexplored population: Chileans of Hispanic descent. Based on the central role of B cells in SLE and on evidence that miRNA expression abnormalities contribute to pathogenic processes in SLE, miRNA microarray analyses were performed on two different B cell subsets purified from the peripheral blood. Our study identified two types of SLE-specific miRNA signatures. We report on lineage-specific miRNAs that are associated with SLE in naive or memory B cell subpopulations and that might serve as biomarkers for disease diagnosis. We also report on miRNAs that are differentially expressed between SLE and LN patients in B cells that might serve as biomarkers for disease type, potentially leading to new therapeutic strategies. Although performed on a small case number that will require further studies, our present work validated two and three miRNAs expression levels in French subjects for both types of SLE-specific miRNA signatures, respectively, which extends our findings to another ethnic group.

Our statistical analysis of the miRNAs differentially expressed between all groups additionally revealed that differences between SLE-affected and unaffected individuals are less important than differences between naive and memory B cell subsets. This observation suggests that B cell subsets from active SLE patients mainly retain several features derived from their normal counterpart B cell subsets, and that only a small number of miRNAs are significantly deregulated in the context of SLE. Such a result is in agreement with a transcriptional study of CD19^+^ B cells that reports weak differences between SLE patients and controls [19] and suggests important similarities at the transcriptomic level for both protein- and miRNA-encoding genes between normal and lupus B cells. We found only 30 miRNAs differentially expressed between all groups, which represent less than 4% of analyzed miRNAs, that discriminate naive CD27^−^ from memory CD27^+^ B cell subpopulations independently of the clinical status of the individuals and/or disease type. In-depth analysis of miRNAs that are differentially expressed in B cell subsets of SLE patients compared with HC, however, point at a different epigenetic regulation in active SLE compared with healthy controls, with only few miRNAs involved (19 miRNAs). The majority displayed increased expression in naive B cells, among which half have been previously involved in the regulation of B cell development in the periphery, such as miRNAs belonging to the miR-17/92 cluster and miR-181 family, as well as miR-141, miR-146a, miR-223, miR-324-3/5p, and miR-342-3/5p [20,21], and reported in PBMCs or *Epstein-Barr* virus-transformed B cell lines from LN patients in African American and European American populations [4]. A variation in the ethnicity does matter as the plasma level of miR-223 was also found increased in a Chinese SLE cohort [22], but decreased in a European population with active lupus nephritis [7]. This variable shall therefore be considered and miRNA patterns identified in previous studies cannot be systematically extrapolated to the Latin American population. Importantly, the differential miRNA expression observed between naive and memory B cells might still be conserved in disease, although reduced, as we observed with miR-223.

Interestingly, when comparing separate B cell subsets isolated from SLE patients with their HC counterparts, the majority of the miRNAs identified in our study were down regulated in the disease setting. These results suggest that these microRNAs may be important in maintaining the naive or memory phenotype in normal B cells, and that their disturbed expression in the respective SLE B cell subsets is associated with lupus. Memory B-cell differentiation and activation are important for quick and long-term adaptive immune responses. These processes are also of interest because memory B-cell abnormalities are associated with autoimmune disorders such as SLE, both in terms of enhanced memory B cell frequencies and disturbed effector functions [10]. Indeed, a quantitative difference in B cell subsets is observed in SLE, as an increase in memory B cells is coupled with a decrease of naive B cells in the peripheral blood of patients. The differential expression patterns of miRNA described here thus add an additional qualitative difference. Interestingly, miR-223 has previously been described as being important in the commitment to B-lymphoid lineages by potentially targeting LMO2 (LIM domain only 2) and MYBL1 (V-Myb Avian Myeloblastosis Viral Oncogene Homolog-Like 1) [20]. Greater expression of miR-223 in the naive B cell stage could inhibit the expression of these transcription factors until the cell is ready to undergo further differentiation into memory B-cell stage. Our data showed that this stage seems to be altered in Latin American SLE patients. 

*In silico* analysis of putative genes targeted by at least two lineage-specific miRNAs deregulated in SLE identified 620 and 42 genes in naive and memory B cells, respectively (Tables S1 and S2). Pathway enrichment analysis with IPA indicated cellular movement, cellular development, and proliferation. Interestingly, the miRNAs that we found deregulated in SLE memory B cells are mainly predicted to target genes previously associated with SLE pathogenesis or B cell biology, and a list of seven genes (*CDKN1A*, *CSF1*, *IFNG*, *JUN*, *MMP9*, *NF-κB1*, and *P53*) showed significant enrichment in inflammatory disease and response, renal inflammation, and renal nephritis canonical pathways. This observation further supports memory B cell abnormalities as playing a critical role in SLE pathogenesis.

## 4. Experimental Section

*Ethics statement:* Fresh peripheral blood samples were obtained from Chilean SLE patients (*n* = 6) of Hispanic descent (according to parents surnames) fulfilling the Systemic *Lupus* Collaborating Clinics SLICC and American College of Rheumatology ACR criteria before therapy. All Chilean SLE patients displayed a severe form of systemic lupus, with (*n* = 4) or without (*n* = 2) lupus nephritis (LN) complication, and relapsed from previous treatments (Table 1). Control subjects (*n* = 4) were sex, age, and ethnically matched healthy individuals (HC), with no history of autoimmune diseases. All SLE patient or HC gave their written informed consent in accordance with procedures approved by the Ethics Committee from Chilean Ministry of Health or the local human ethics committee (Comité de Protection des Personnes Sud Méditerrannée IV: ID RCB 2008-A01087-48). The mean age of French SLE patients (*n* = 4) was 30.25 ± 12.70 years and the SLE disease activity index (SLEDAI) was 9.5 ± 5.0. Mononuclear cells from peripheral blood were separated by Ficoll-Paque PLUS density gradient (GE Healthcare, Uppsala, Sweden). Total CD19^+^ cells were isolated by the use of B cell isolation microbeads kit (Milteny Biotec, Paris, France). Naive and memory B cells were sorted on the basis of the CD27 surface marker using the CD27 microbeads kit (Milteny Biotec, Paris, France). Purity of the CD27^+^ and CD27^−^ B cell subsets was monitored by FACS analysis and was 92%–95% and 98%–99%, respectively.

*miRNA real-time quantitative PCR.* Total RNAs, including small RNAs, were extracted using a miRNeasy Mini Kit with a Qiacube (QIAGEN, Courtaboeuf, France) according to the manufacturer’s instruction. RNA concentration, purity, and quality were assessed using the 2100 Bioanalyzer assay (Agilent, Les Ulis, France), and according the criteria of the Minimum Information for Publication of Quantitative Real-Time PCR Experiments MIQE guidelines, only samples with a RIN >8 were used. Total RNAs (15 ng) were converted to cDNAs using Megaplex™ RT Primers that contain a pool of 750 individual miRNA-specific primers and TaqMan MicroRNA Reverse Transcription kit (Applied Life Technologies, Saint Aubin, France). A pre-amplification step using Megaplex™ PreAmp Primers and TaqMan^®^ PreAmp Master Mix (Applied LifeTechnologies, Saint Aubin, France) was performed. miRNA profiling was performed using the TaqMan^®^ Human MicroRNA Array Cards v3.0 (Applied Life Technologies, Saint Aubin, France). The 384-well format TLDAs were run on an ABI 7900HT fast real-time PCR system (Applied Life Technologies, Saint Aubin, France). RT-qPCR raw data were analyzed using SDS 2.3 and RQ Manager Software (Applied Biosystems, Saint Aubin, France). MiRNA values of CD27^−^ and CD27^+^ cells for each sample were normalized to the mean Ct value of all expressed miRNAs and 2 snoRNAs RNU44 and RNU48. Relative miRNA expression was calculated using the comparative threshold cycle (*C*_t_) method. *C*_t_ values <30 were selected for volcano plot visualization. Using a fold change (FC) ± 1.8, we selected a set of miRNAs that were analyzed individually using a conventional one-way ANOVA test. Finally, we used the ExpressionSuite software v1.0.3 to build a heatmap for miRNAs significantly up- or down-regulated between groups of comparison.

*Statistical analysis.* GraphPad prism software was used to perform an ordinary one-way ANOVA test for multiple group comparisons, followed by a Tukey’s multiple comparisons post-test when 3 or more samples per group were tested. *p* values less than 0.05 were considered statistically significant. 

## 5. Conclusions

While a human miRNome analyzing different stages of B cell development has already been published [23], a miRNomic study tracking miRNA expression in B cell subsets along with lupus development remains to be done. In this line, the comparison of miRNA expression in the different B cell subsets performed here represents an important approach enabling a deeper characterization of pathogenic processes in SLE pathogenesis and, hence, further facilitating our understanding of such a complicated disease. The subset-specific miRNA profiling that we identified could also be particularly interesting to monitor variations following a B cell depleting therapy, such as the use of anti-CD20 antibodies. Indeed, these treatments induce incomplete B cell depletion in some patients, contributing to poor clinical responses, and the mechanisms of resistance remain elusive. Finally, while the characterization of miRNA expression patterns performed previously in SLE patients can be of potential diagnostic use, discoveries in B lymphocyte subset-specific miRNA expression profiles during disease progression can provide a deeper insight into SLE immune-pathogenesis.

## Supplementary Materials

Supplementary materials can be found at http://www.mdpi.com/1422-0067/16/08/16953/s1.
